# To the Editors of the Medical and Physical Journal

**Published:** 1807-06

**Authors:** Robert Scammell

**Affiliations:** Plymouth


### *To the Editors of the Medical and Physical Journal.*

GENTLEMEN,

THOUGH cases in themselves extraordinary, and of rare occurrence, may not be said to be of practical utility, yet they are certainly of too important a nature to be entirely buried in oblivion; to prevent which, you will oblige me by inserting the following in your very useful periodical publication.

In March last, I was requested by a young man, Robert Bartlett, about 18 years of age, to give my opinion on an eruptive disorder under which he then laboured, and previous to which he had had very considerable fever; a slight inspection was quite sufficient to convince me he had the small-pox, and I gave him my opinion to that effect; he appeared greatly surprized, thanked me, but seemed not altogether satisfied; I told his mother it certainly was the small-pox; she remarked, it could not be so; it was absolutely impossible. This united obstinacy naturally excited my particular attention, and I enquired of her whence arose her want of belief; she replied, she understood no one ever had the small-pox twice; her son had been inoculated when a few months old, was very feverish, his arm greatly inflamed, and had several small-pox, which were very large and filled in a proper manner, which were fully confirmed by the neighbours. This was her reason for considering her son's complaint to be of a different nature. I could not convince her; only observing, that a short time would prove the truth or fallacy of my opinion.

A few days after I called on her, and found her converted, as the case admitted then of no doubt, the pustules being very numerous and large.

It happened that this young man, from an absolute sense of security from the contagion of small-pox, had been at times exposed to its utmost virulence.

He got this disorder in consequence of a child dying in the confluent species, and being a carpenter, his situation obliged him to assist in the last ceremonies.

I am, &c.

ROBERT SCAMMELL.

*Plymouth, May 9, 1807.*

